# Positive association between chronic hepatitis B virus infection and anemia in pregnancy in Southern China

**DOI:** 10.1038/s41598-024-84927-7

**Published:** 2025-01-15

**Authors:** Renliang Huang, Zhe Lu, Xinze Li, Duo Zhou, Jing Xu, Dan Lin, Yunxue Fu, Yan Liang, Xuexia Li, Frank Petersen, Qiaomiao Zhou, Xinhua Yu

**Affiliations:** 1 Hainan Women and Children’s Medical Center, Haikou, 571199 Hainan China; 2 NHC Key Laboratory of Tropical Disease Control, School of Tropical Medicine, Hainan Medical University, Haikou, Hainan China; 3 Priority Area Chronic Lung Diseases, Research Center Borstel, Leibniz Lung Center, Borstel, 23845 Germany

**Keywords:** Anemia in pregnancy, Hepatitis B virus, Infection, Observational study, Association, Health care, Public health, Epidemiology

## Abstract

This observational investigation aimed to explore potential risk factors for anemia in pregnancy. Firstly, a cross-sectional study was conducted, encompassing a review of clinical data of 43,201 pregnant women admitted to the Hainan Women and Children’s Medical Center between January 2017 and December 2020. Comparison between women with and without anemia in pregnancy revealed significant differences between the two groups concerning age, gestational diabetes, hypothyroidism, hyperthyroidism, chronic hepatitis B virus infection, syphilis infection, and human immunodeficiency virus infection. Multivariable logistic regression analysis showed that chronic hepatitis B virus infection was significantly associated with anemia during pregnancy (AOR   2.97, 95% CI 2.57–3.44, *p* < 0.0001). Subsequently, a retrospective cohort comprising 86 cases with chronic hepatitis B virus infection and 129 control subjects recruited from the Hainan Women and Children’s Medical Center from November 2021 and January 2023 was examined. Results of the examination revealed a corroborative association between chronic hepatitis B virus infection and anemia in pregnancy (OR  2.13, 95% CI 1.20–3.79, *p* = 0.0092), particularly manifesting in the third trimester of gestation. Further analysis unveiled distinctive hematological alterations among cases with chronic hepatitis B virus infection, characterized by diminished erythrocyte size and reduced levels of corpuscular hemoglobin. Collectively, these findings underscore a positive association of chronic hepatitis B virus infection with anemia during pregnancy.

## Introduction

Anemia in pregnancy, characterized by a hemoglobin (Hb) concentration below 11 g/dl in pregnant women, affects nearly 50% of pregnancies on a global scale^1^. Its association with maternal morbidity, mortality, and adverse fetal outcomes underscores its status as a significant public health concern worldwide, particularly prevalent in developing nations^1,2^. The etiology of pregnancy-related anemia is multifaceted, with iron deficiency representing a primary causal factor alongside with various other contributors including malnutrition, thalassemia, and infectious diseases such as hookworm infection or malaria^3–7^. In this observational study, we sought to identify potential risk factors associated with anemia during pregnancy. To achieve this, we conducted both a cross-sectional analysis and a retrospective cohort study, with anemia in pregnancy defined as the primary outcome variable.

## Methods

### Subjects and data collection

The cross-sectional study encompassed a review of demographic and clinical records pertaining to pregnant women admitted to the Hainan Women and Children’s Medical Center (HNWCMC) between January 2017 and December 2020. Exclusion criteria were applied to eliminate patients under 18 years old, cases of stillbirth, and individuals admitted for reasons other than delivery. Anonymized data including age, diagnosis of gestational diabetes, anemia in pregnancy, hypothyroidism, hyperthyroidism, chronic hepatitis B virus (HBV) infection, syphilis infection, and human immunodeficiency virus (HIV) infection, were extracted from the electronic medical records system. All patients were well characterized for all abovementioned clinical and demographic data. To identify factors associated with anemia, anemia in pregnancy was treated as the dependent variable, while age, gestational diabetes, hyperthyroidism, chronic HBV infection, syphilis infection, and HIV infection were considered as independent variables.

The retrospective cohort included 86 HBV cases and 129 controls who were admitted to the HNWCMC between November 2021 and January 2023. Serum samples were prepared by allowing 5 ml peripheral blood to clot at room temperature for 30 min. Afterwards, the samples were centrifuged at 3000 rpm for 10 min, and supernatant was collected for further analysis. Serum samples were utilized for serological assessment to determine the status of HBV infection. The presence of HBsAg, anti-HBsAg, HBeAg, anti-HBeAg, and anti-HBcAg was determined utilizing the iFlash 3000 chemiluminescence immunoassay analyzer in conjunction with the iFlash anti-HBcAg kit (YHLO Biotech, Co., Ltd. Shenzhen, China). Chronic HBV infection was defined as the presence of HBsAg for at least six months during the pregnancy. Serum levels of alanine transaminase (ALT) and aspartate aminotransferase (AST) were quantified utilizing specific kits (XinChuangYuan Biotech, Co., Ltd. Beijing, China), adhering strictly to the manufacturers’ instructions. The 86 HBV cases tested positive for HBsAg either before pregnancy (*n* = 7) or during the first trimester of pregnancy (*n* = 79), and all retained positive HBsAg results in a subsequent test conducted at least 6 months later. In contrast, the 129 controls were negative for HBsAg throughout the pregnancy. Hematological parameters for all HBV cases and controls were assessed during the first, second, and third trimesters to diagnose anemia in pregnancy using either the BC-6100Plus Analyzer (Mindray, USA) or the Sysmex XN-1000 Analyzer (Sysmex, Japan). Parameters pertaining to red blood cells, encompassing red blood cell count (RBC), hemoglobin concentration (HGB), hematocrit (HCT), mean corpuscular volume (MCV), mean corpuscular hemoglobin (MCH), mean corpuscular hemoglobin concentration (MCHC), red blood cell distribution width-standard deviation (RDW-SD), and RDW-coefficient of variation (RDW-CV), were subjected to analysis. The diagnosis of anemia in pregnancy was established in accordance with World Health Organization (WHO) criteria, defining anemia as hemoglobin levels below 11.0 g/dL during the pregnancy^8^. All studied subjects in the retrospective cohort were characterized for RBC-associated parameters in all three trimesters of pregnancy and assessed for chronic HBV infection.

This study was performed in line with the principles of the Declaration of Helsinki^9^. Approval for the study protocol was obtained from the Ethics Committee of the HNWCMC (Approval No.: HNWCMC-2023-80). Given the exclusively anonymized nature of the data utilized in this study and no more than minimal risk to the subjects, the requirement for informed consent was waived by the Institutional Review Board.

### Detection of thalassemia genetic variations

For genomic analysis, DNA extraction from peripheral blood leukocytes was performed utilizing the Nucleic Acid Extraction or Purification Kit (Guangzhou Ruineng Medical Technology Co., Ltd. Guangzhou, China), following the manufacturer’s protocols. Common thalassemia mutations were identified utilizing the polymerase chain reaction-reverse dot blot hybridization technique, as previously described^10^.

### Statistical analyses

All statistical analyses were meticulously conducted using GraphPad Prism Software (version 10.0.2). The Kolmogorov-Smirnov test was utilized to assess the normal distribution of variables. The student’s t-test was utilized to assess statistical significance for normally distributed quantitative data, while the Mann–Whitney U test was employed for non-normally distributed data. For binary variables, the chi-squared test or Fisher’s exact test was employed to ascertain statistical significance and odd ratios (ORs). Additionally, multivariable logistic regression analysis was executed to evaluate the predictive capacity of multiple independent variables on a binary outcome, and adjusted odd ratios (AORs) were calculated to quantify these associations. A *p*-value less than 0.05 was considered statistically significant.

## Results

### Association between chronic HBV infection and anemia in pregnancy in a cross-sectional study

A total of 43,201 pregnant women admitted to HNWCMC between January 2017 and December 2020 were screened. After applying the exclusion criteria, 38,180 pregnant women, with a mean age of 29.3 years, were included in the cross-sectional study (Supplementary Fig. 1, Table 1). Among the 38,180 cases, 7357 (19.27%) were diagnosed with anemia in pregnancy, 2548 (6.67%) developed gestational diabetes, 344 (0.90%) showed hypothyroidism, and 60 (0.16%) exhibited hyperthyroidism. The frequencies of chronic HBV infection, syphilis infection, and HIV infection were recorded at 2.10, 0.21, and 0.026%, respectively. (Table 1)

**Table 1:** Demographic and clinical information of patients in the discovery phase of the study.

	Pregnant women (*n* = 38,180)
Age in years (mean ± SD)	29.3 ± 4.8
Anemia in pregnancy	7357 (19.27%)
Gestational diabetes	2548 (6.67%)
Hypothroidism	344 (0.90%)
Hyperthyroidism	60 (0.16%)
Chronic HBV infection	803 (2.10%)
Syphilis infection	80 (0.21%)
HIV infection	10 (0.026%)

*SD* standard deviation, *HBV* hepatitis B virus, *HIV* human immunodeficiency virus.

To determine potential risk factors for anemia in pregnancy, the pregnant women were stratified into two groups: 7,353 cases with anemia and 30,823 individuals without anemia. Subsequent analysis revealed significant disparities between the two groups concerning age, gestational diabetes, hypothyroidism, hyperthyroidism, chronic HBV infection, syphilis infection, and HIV infection. Multivariable logistic regression analysis was then employed, revealing that advanced maternal age (AOR  1.01, 95% CI 1.00-1.01, *p* < 0.0001), gestational diabetes (AOR 2.19, 95% CI 2.00-2.39, *p* < 0.0001), hypothyroidism (AOR   2.56, 95% CI 2.05–3.19, *p* < 0.0001), hyperthyroidism (AOR  1.95, 95% CI 1.12–3.30, *p* = 0.015), chronic HBV infection (AOR  2.97, 95% CI 2.57–3.44, *p* < 0.0001), and syphilis infection (AOR   1.74, 95% CI 1.06–2.79, *p* = 0.025) were each associated with an elevated risk of anemia in pregnancy (Table 2). Consequently, in addition to reaffirming the previously established associations between anemia in pregnancy and gestational diabetes^11^, hypothyroidism and hyperthyroidism^12^, this cross-sectional study revealed a robust positive association between chronic HBV infection and anemia in pregnancy.

**Table 2:** Factors associated with anemia in pregnancy in the retrospective cross-sectional study.

	Anemia (*n* = 7,357)	Without anemia (*n* = 30,823)	*p* values	Multivariable logistic regression analysis			
				|Z|	VIF	AOR (95% CI)	*p* values
Age in years (mean ± SD)	29.6 ± 4.5	29.3 ± 4.9	< 0.0001	2.09	1.01	1.01 (1.00–1.01)	0.0366
Gestational diabetes	891 (12.11%)	1657 (5.38%)	< 0.0001	17.50	1.02	2.19 (2.00–2.39)	< 0.0001
Hypothroidism	138 (1.88%)	206 (0.67%)	< 0.0001	8.35	1.00	2.56 (2.05–3.19)	< 0.0001
Hyperthyroidism	22 (0.30%)	38 (0.12%)	0.0006	2.42	1.00	1.95 (1.12–3.30)	0.0154
Chronic HBV infection	357 (4.85%)	446 (1.45%)	< 0.0001	14.75	1.01	2.97 (2.57–3.44)	< 0.0001
Syphilis infection	25 (0.34%)	55 (0.18%)	0.0065	2.24	1.00	1.74 (1.06–2.79)	0.0248
HIV infection	5 (0.068%)	5 (0.016%)	0.0137	1.93	1.00	3.55 (0.93–13.25)	0.054

*SD* standard deviation, *HBV* hepatitis B virus, *HIV* human immunodeficiency virus, *VIF* variance inflation factor, *AOR* adjusted odd ratio.

We also investigated the association between chronic HBV infection and other pregnancy-related complications. As summarized in Supplementary Table 1, chronic HBV infection was positively associated with gestational diabetes and hypothyroidism, but no significant association was found between chronic HBV infection and hyperthyroidism. Furthermore, while both chronic HBV infection and gestational diabetes were independently associated with anemia during pregnancy, no cumulative effect of these factors on anemia was observed (Supplementary Table 2).

### Association between chronic HBV infection and anemia in pregnancy in a retrospective cohort

To confirm the association between chronic HBV infection and anemia in pregnancy, a retrospective cohort study was subsequently conducted. The cohort comprised 86 pregnant women who tested positive for HBsAg either before conception or during the first trimester, while 129 age-matched pregnant women, testing negative for HBsAg, constituted the control group. The HBV cases exhibited markedly elevated serum levels of alanine transaminase (ALT) and aspartate aminotransferase (AST), consistent with the profile of chronic HBV infection (Table 3).

**Table 3:** Association of chronic HBV infection with anemia in pregnancy in the validation retrospective cohort.

	Healthy controls (*n* = 129)	HBV case (*n* = 86)	OR (95% CI)	*p* values
Age in years, (mean ± SD)	30.2 ± 3.7	30.9 ± 4.0		n.s.
ALT (U/L)*, median (min., max.)	12 (5, 97)	21 (6, 325)		< 0.0001
AST (U/L)*, median (min., max.)	16 (10, 49)	21 (13, 277)		< 0.0001
Anemia in the first trimester, n (%)	6 (4.7%)	11 (12.8%)	3.01 (1.07–8.47)	0.0303
Anemia in the second trimester, n (%)	54 (41.9%)	43 (50%)	1.39 (0.80–2.40)	n.s.
Anemia in the third trimester, n (%)	53 (41.1%)	52 (60.5%)	2.19 (1.25–3.83)	0.0054
Anemia in any trimester, n (%)	67 (51.9%)	60 (69.8%)	2.13 (1.20–3.79)	0.0092

*ALT* alanine transaminase, *AST* aspartate aminotransferase, *HBV* hepatitis B virus, *OR* odd ratio.*Data derived from 118 controls and 83 HBV cases.

Throughout the entirety of pregnancy, both HBV cases and controls were monitored for the onset of anemia. During the first trimester, 12.8% (11 out of 86) of HBV cases developed anemia, a proportion significantly higher than the 4.7% observed in control subjects (OR 3.01, 95% CI 1.07–8.47, *p* = 0.303). Interestingly, no discernible difference in the prevalence of anemia emerged between the two groups during the second trimester (50% vs. 41.1%, OR 1.39, 95% CI 0.80–2.40, *p* = 0.24). However, a notable discrepancy in the prevalence of anemia resurfaced in the third trimester, with 60.5% of HBV cases and 41.1% of control subjects exhibiting anemia (OR 2.19, 95% CI 1.25–3.83, *p* = 0.0054). Cumulatively considering anemia across any trimester, 69.1% of HBV cases and 51.9% of control subjects were affected during pregnancy (OR 2.13, 95% CI 1.20–3.79, *p* = 0.0092), thereby corroborating the positive association between chronic HBV infection and anemia in pregnancy (Table 3).

### No correlation between chronic HBV infection and thalassemia

To ascertain whether the observed association between chronic HBV infection and anemia in pregnancy might be confounded by the presence of thalassemia, a prevalent risk factor for anemia in pregnancy in the Hainan population^2,10^, a genetic analysis was conducted. Specifically, the cohort of 86 HBV cases and 129 controls was genotyped for common genetic variations of α and β thalassemia. Table 4 presents the prevalence of carriers of thalassemia genetic variations among HBV cases, which is 32.6% (28 out of 86), similar to the prevalence observed among the control group (32.6%; 42 out of 129). When the patients were subdivided based on specific subtypes of thalassemia, the stratified analysis revealed no significant association between chronic HBV infection and any subtype of thalassemia. Consequently, these findings suggest that there is no discernible association between HBV infection and thalassemia.

**Table 4:** Analysis of the relationship between HBV infection and thalassemia mutations in the retrospective cohort used in the validation phase.

	Healthy controls (*n* = 129)	HBV infection (*n* = 86)	OR (95% CI)	*P* values
Non-thalassemia carrier	87 (67.4%)	58 (67.4%)		
Thalassemia carrier (total)	42 (32.6%)	28 (32.6%)	1.00 (0.55–1.76)	n.s.
α-thalassemia silent carrier	24 (18.6%)	20 (23.3%)	1.25 (0.63–2.42)	n.s.
α-thalassemia minor	17 (13.1%)	7 (8.1%)	0.62 (0.24–1.67)	n.s.
β-thalassemia minor	1 (0.01%)	0 (0%)	0.00 (0.00-13.66)	n.s.
α-thalassemia minor + β-thalassemia minor	0 (0%)	1 (0.01%)	∞ (0.16-∞)	n.s.

*HBV* hepatitis B virus, *OR* odd ratio.

### Association between chronic HBV infection and erythrocytes parameters

**Fig. 1:**
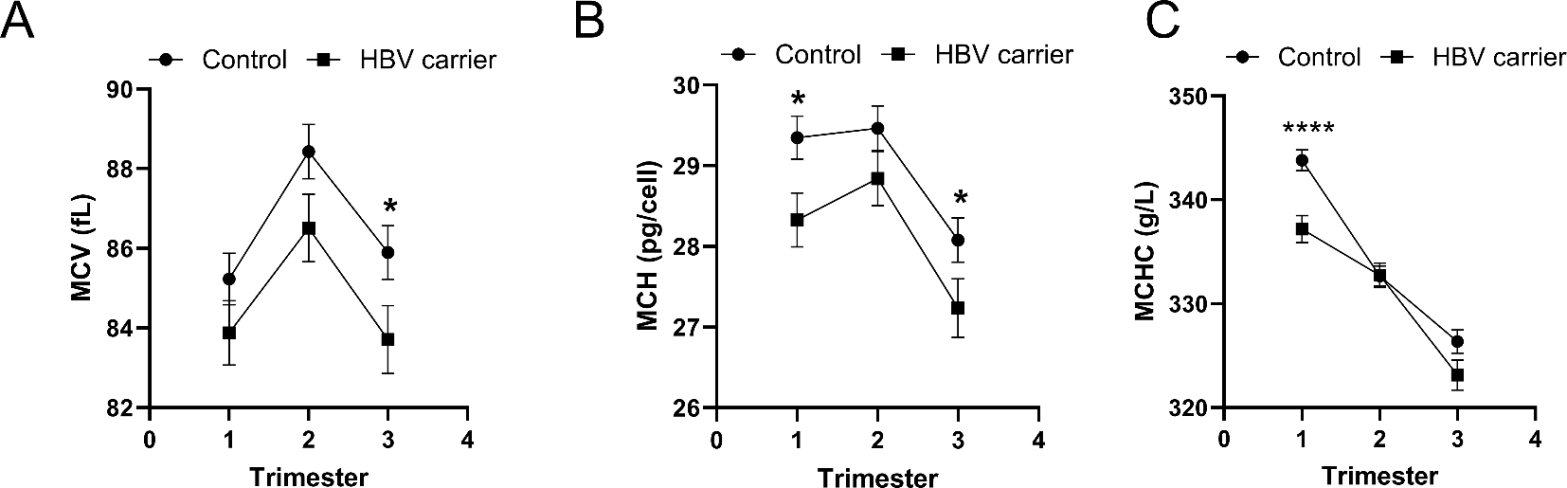
Association between chronic hepatitis B virus (HBV) infection and MCV, MCH and MCHC. Comparison of mean corpuscular volume (MCV) (**A**), mean corpuscular hemoglobin (MCH) (**B**), and mean corpuscular hemoglobin concentration (MCHC) (**C**) between HBV cases and control subjects during three trimesters of pregnancy. Data are presents as mean ± SEM (standard error of mean). Statistical significance between control subjects and HBV cases was determined using unpaired students T test. *p < 0.05, ****p < 0.0001.

To gain insight into the positive association between chronic HBV infection and anemia in pregnancy, we explored the relationship between the presence of HBsAg and various erythrocyte parameters beyond HGB, including RBC, HCT, MCV, MCH, MCHC, RDW-SD and RDW-CV. No significant disparities were observed between HBV cases and controls in RBC, HCT, RDW-SD, or RDW-CV across any trimester. However, discernible differences were noted between the two groups in MCV, MCH, and MCHC. Although both HBV cases and control subjects exhibited similar trajectories in these parameters throughout pregnancy, HBV cases consistently demonstrated a tendency toward decreased MCV, MCH, and MCHC compared to control subjects. Specifically, significant differences in MCV during the third trimester, MCH in the first and third trimesters, and MCHC in the first trimester were observed (Fig. 1, Supplementary Table 3).

## Discussion

In the retrospective cross-sectional study encompassing 38,180 pregnant women, we observed a prevalence of anemia in pregnancy amounting to 19.27%. Notably, this finding aligns closely with the prevalence (17.78%) reported in a previous study involving 18 million Chinese pregnant women nationwide^13^. Such congruence lends credence to the reliability of our findings. However, in the retrospective cohort recruited from the same center in this study, the prevalence of anemia in pregnancy exceeded 50%. This substantial disparity is likely attributable to more meticulous follow-up procedures implemented in the retrospective cohort compared to the cross-sectional study. This discrepancy underscores a notable limitation inherent in retrospective cross-sectional studies, namely the potential for underestimation of prevalence due to less rigorous surveillance.

Notably, the cross-sectional study revealed a positive association of chronic HBV infection with anemia in pregnancy, and the association was confirmed in a retrospective cohort. Consequently, through the amalgamation of a cross-sectional study and a retrospective cohort analysis, this investigation furnishes compelling evidence supporting an association between chronic HBV infection and an elevated risk of anemia in pregnancy. Both anemia during pregnancy and hepatitis B virus (HBV) infection pose significant public health challenges in the developing world. However, the potential link between the two pathogenic conditions remains uncertain. Recent observational studies have yielded conflicting evidence on this matter. By reviewing of medical records encompassing 16,727 pregnant women, Deng et al. reported a higher prevalence of anemia among HBsAg cases compared to controls in southern China^14^, suggesting a positive association between HBV infection and anemia in pregnancy. However, such an association was not confirmed by two other retrospective observational studies, each with comparable sample sizes^15,16^. Therefore, the results of our study resolve the discordance evident in prior observational studies concerning this issue^14–16^. One possible explanation for the discrepancy might be missing data, which is a common limitation of retrospective studies^17^. Consequently, the findings of this study advocate for a cautious interpretation of observations derived from cross-sectional studies. Ideally, such findings should be validated through complementary observational studies to ensure robustness and reliability.

Despite the significant reduction in the prevalence of HBV infection following the introduction of the HBV vaccine, over 1.5 million new HBV infections continue to occur annually^18,19^. This persistence is largely attributed to the substantial variation in HBV vaccination coverage globally^20^. It is estimated that over 300 million individuals across the world live with chronic HBV infection^19^. Additionally, approximately 4.5 million women with chronic HBV infection give birth annually^21^. Consequently, identifying the association between chronic HBV infection and anemia in pregnancy carries substantial public health implications, particularly in developing countries.

Given that HBV infection often precedes the development of anemia during pregnancy, the observed positive association between the two conditions suggests that HBV infection may contribute to the development of anemia in pregnancy. This potential relationship and its underlying mechanisms warrant further investigation. Notably, subsequent analyses revealed distinct hematological characteristics, notably reduced erythrocyte size and corpuscular volume in HBV cases compared to control subjects. This finding suggests that chronic HBV infection may confer the risk of anemia in pregnancy by influencing the volume of erythrocytes and levels of corpuscular hemoglobin, rather than impacting the quantity or size variability of erythrocytes. Iron deficiency stands as the most prevalent cause of microcytic and hypochromic anemia^22^. While thalassemia represents another common etiology of microcytic and hypochromic anemia, particularly prevalent in tropical and subtropical regions^23^. Given the exclusion of an association between chronic HBV infection and thalassemia in our study, it is conceivable that HBV infection may elevate the risk of anemia in pregnancy by influencing iron metabolism. This hypothesis finds support in observations of iron metabolism disorders occurring in individuals with HBV-related liver diseases^24^. Consequently, the evaluation of iron supplementation necessity for HBV cases during pregnancy may emerge as a clinically consideration.

Interestingly, the current study confirms two previous established associations: the association between gestational diabetes and anemia in pregnancy^11^ and the association between chronic HBV infection with gestational diabetes^25,26^. Given that both chronic HBV infection and gestational diabetes are positively associated with anemia during pregnancy, it is conceivable that these factors might have an accumulative or synergistic effect on anemia. However, the present study found no evidence of such an accumulative or synergistic effect. This finding suggests the presence of an unknown interaction among these three conditions, which warrants further investigation.

This study has three notable limitations. Firstly, although HBV infection preceded the development of anemia during pregnancy in the majority of HBV cases in the retrospective cohort, for the small number of cases where anemia developed in the first trimester, it is challenging to determine whether HBV infection or anemia occurred first. This limitation may affect the validity of the findings from the retrospective cohort. Secondly, since the observational studies were conducted at a single center in Hainan, China, caution is needed when generalizing the results to other populations. Thirdly, age is a significant factor that could influence pregnancy-related complications, including anemia^27,28^, as reflected in our cross-sectional study. Given that the median age of the participants was approximately 30 years, the findings may not be generalizable to populations of different age groups. Further research is needed to confirm the association across diverse age groups.

In summary, our study delineates a discernible association between chronic HBV infection and an escalated susceptibility to anemia in pregnancy, as evidenced by rigorous cross-sectional and retrospective cohort investigations. These findings hold public health implications for clinical practice, particularly for developing countries with high prevalence of HBV infection.

## Electronic supplementary material

Below is the link to the electronic supplementary material.

Supplementary Material 1

## Data Availability

Data is provided within the manuscript.
