# Supplementary material for: Positive association between chronic hepatitis B virus infection and anemia in pregnancy in Southern China
Source: Sci Rep. 2025 Jan 15;15:1980. doi: 10.1038/s41598-024-84927-7 (PMC11732989; doi:10.1038/s41598-024-84927-7)
Supplement: Supplementary file 1 — Supplementary Material 1 [file 41598_2024_84927_MOESM1_ESM.docx]

**Supplementary information**

**
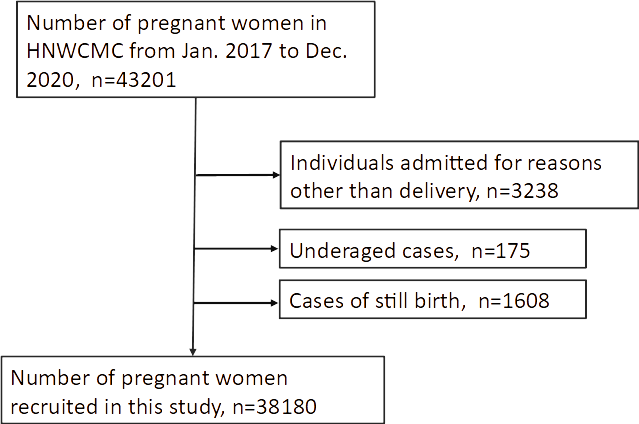
**

# Supplementary Figure 1. Flow diagram describing recruitment of participants to the cross-sectional study. HNWCMC. Hainan Women and Children’s Medical Center.

**Supplementary Table 1. Association between chronic HBV infection and pregnancy-related complications**

|  | **With chronic HBV infection**  **(n=802)** | **Without chronic HBV infection (n=37,378)** | ***p* values** |
| --- | --- | --- | --- |
|  |  |  |  |
| Age in years (mean±SD) | 30.4±4.2 | 29.3±4.8 | <0.0001 |
| Anemia in pregnancy | 356 (44.39%) | 7001 (18.73%) | <0.0001 |
| Gestational diabetes | 201 (25.06%) | 2347 (6.28%) | <0.0001 |
| Hypothroidism | 14 (1.75%) | 330 (0.88%) | 0.0204 |
| Hyperthyroidism | 3 (0.37%) | 57 (0.15%) | 0.1319 |

SD. Standard deviation; HBV. hepatitis B virus.

**Supplementary Table 2. Relationship between chronic HBV infection, gestational diabetes and anemia in pregnancy**

|  | **Prevalence of anemia** | **OR (95% CI)*** | ***p* values*** |
| --- | --- | --- | --- |
|  |  |  |  |
| HBsAg-GDM- (n=28660) | 6199 (21.62%) |  |  |
| HBsAg-GDM+ (n=2346) | 801 (34.14%) | 1.88 (1.72-2.06) | <0.0001 |
| HBsAg+GDM- (n=601) | 267 (44.42%) | 2.89 (2.46-3.41) | <0.0001 |
| HBsAg+GDM+ (n=202) | 90 (44.54%) | 2.91 (2.20-3.84) | <0.0001 |

GDM. gestational diabetes mellitus; OR. Odd ratio. *OR and *p* values were calculated as compared with subjects without chronic HBV infection and without gestational diabetes (HBsAg-GDM-).

**Supplementary Table 3. Chronic HBV infection and RBC related parameters during the pregnancy**

|  | **First trimester** | | **Second trimester** | | **Third trimester** | |
| --- | --- | --- | --- | --- | --- | --- |
|  | Control (n=129) | HBV carrier (n=86) | Control (n=129) | HBV carrier (n=86) | Control (n=129) | HBV carrier (n=86) |
| RBC (10^9^/ml) | 4.32±0.43 | 4.38±0.48 | 3.82±0.39 | 3.86±0.43 | 3.96 ±0.39 | 4.00±0.46 |
| HCT (%) | 36.6±2.5 | 36.5±3.2 | 33.6±2.9 | 33.2±2.6 | 33.8±2.8 | 33.2±2.5 |
| MCV (fL) | 85.2±7.4 | 83.9±7.5 | 88.4±7.8 | 86.5±7.8 | 85.9±7.7 | 83.7±8.0* |
| MCH (pg/cell) | 29.4±3.1 | 28.3±3.1* | 29.5±3.1 | 28.8±3.1 | 28.1±3.1 | 27.2±3.4* |
| MCHC (g/L) | 343.8±11.4 | 337.2±12.0**** | 332.7±11.3 | 332.7±10.9 | 326.4±12.9 | 323.1±13.4 |
| RDW-SD (%) | 41.9±3.9 | 42.0±4.1 | 43.7±2.7 | 43.2±4.1 | 44.5±3.3 | 44.0±3.4 |
| RDW-CV (%) | 13.5±1.8 | 13.7±1.7 | 13.5±1.4 | 13.6±1.4 | 14.1±1.5 | 14.4±1.8 |

HBV. hepatitis B virus; RBC. red blood cell count; HCT. Hematocrit; MCV. mean corpuscular volume; MCH. mean corpuscular hemoglobin; MCHC. mean corpuscular hemoglobin concentration; RDW-SD. red blood cell distribution width-standard deviation; RDW-CV. red blood cell distribution width-coefficient of variation. Statistical significance between control subjects and HBV carriers was determined using unpaired students T test. **p*<0.05, *****p*<0.0001
